# Report of Vivisections, Etc.

**Published:** 1871-04

**Authors:** F. L. Wadsworth

**Affiliations:** Chicago


ARTICLE II.—*Report of Vivisections, Etc.* Performed by Prof. J. W. FREER, M.D., before the Alumni of Rush Medical College, Chicago. By F. L. WADSWORTH, M.D., Chicago.

At the close of the formal exercises of the Alumni meeting of Rush Medical College, Feb. 1st, 1871, the audience repaired to the upper lecture room of the college building, to witness an exposition of scientific vivisection, by Prof. J. W. Freer, M.D., Prof. of Physiology and Microscopic Anatomy.

It being the custom of Prof. Freer to fully illustrate his course of lectures on Physiology by vivisections, thereby exposing the *living organs* to view, and affording opportunity to observe and demonstrate the functions of various organs, there are continually under observation several animals upon which operations have been performed.

On this occasion the first animal exhibited by Prof. Freer, was one upon which a biliary fistula had been produced. An incision had been made at the median line of the abdomen, extending from the ensiform cartilage to the umbilicus, some three or four inches, exposing the lower border of the liver, duodenum, etc. The common duct had been occluded by a ligature, cutting off the flow of bile to the duodenum, the fundus of the gall bladder had been opened, and one end of a silver tube secured therein by ligatures; the other end, passing through the incision in the abdomen, delivered the bile outside the animal's body. The incision had closed perfectly around the tube, by granulations, holding it securely in its place.

At the date of the operation, Dec. 20th, 1870, the weight of the animal was 15 lbs. 7 oz. avoirdupois, and he ate, daily, an average of 4043 grains of meat. He recovered from the effects of the operation in five days, after which he ate, daily, an average of 4550 grains of meat, while the increase in amount of water drank was fully one hundred per cent. The bile discharged more or less freely from the tube continually; the maximum amount collected during periods of observation being six fluid drams in twelve hours.

At the date of the exhibition—forty-two days after the operation—although the animal's daily rations of meat had been increased by 507 grains, his weight had diminished to 11 lbs. 14 oz.—a loss of 3 lbs. 9 oz., one-quarter of his original weight. These data coincide fully with those obtained by Louget, Bidder and Schmidt, Schwann, Bloudlot and others under similar circumstances.

Observations showed that during the period named, subsequent to the operation, the animal was exceedingly sensitive to cold; that depression by cold diminished the flow of bile; that artificial warmth increased the flow of bile perceptibly; that water taken after fasting, and when the bile was scanty, speedily augmented the amount discharged.

Prof. Freer remarked, that, from these observations, it appeared that bile was not a purely excrementitious substance, but that it played a very important part in the process of digestion. It produces with fats a most perfect emulsion, in which condition the fatty portions of food are absorbed, and brought under the influence of vital force. The absence of bile in the alimentary canal results in rapid emaciation of the adipose tissue, and a great decline in the heat-producing capacities of the body.

The next animal exhibited was a large dog, weighing some 40 or 50 lbs., on which an operation for gastric fistula had been performed. An incision about three inches long had been made at the median line of the abdomen, exposing the anterior surface of the stomach, near the inferior border. The serous surface of this organ was then brought in contact with the abdominal wall and secured by sutures to the lips of the incision, after which the wall of the stomach was pierced by a bistoury, and an opening made about one inch in length, corresponding in direction with the incision in the abdominal pariety. The canula, an instrument $2\frac{1}{4}$ inches in diameter, including flange, and $1\frac{1}{2}$ inches long, was then inserted into this opening, readily, owing to the elasticity of the stomach tissues, which, after insertion, tightly grasped the body of the instrument, preventing hemorrhage and leakage of the stomach fluids into the peritoneal cavity. The incision in the abdominal wall was then carefully closed around the canula, which completed the operation.*

The size of the canula, ($1\frac{1}{4}$ inch diameter of body,) allowed free inspection of the mucous surface of the stomach, which on this occasion poured out gastric juice freely when gently stimulated by the touch of a glass rod. Prof. Freer showed, by experiment, that gastric juice added to milk immediately precipitated the casein.

Next a black and tan terrier, weighing about 25 lbs., was put upon the table and anæsthetized with chloroform. The right common carotid artery and jugular vein were exposed. Into the former a glass canula was inserted and secured by a ligature, and into the latter the detached nozzle of the transfusion syringe was similarly adjusted. The blood of the animal was then allowed to flow from the artery into a vessel where it was defebrinated by whipping, and kept at 100° Fahrenheit, by means of a water bath. The bleeding continued until no more blood would flow, even with the animal's hind quarters raised far above the table upon upon which the head rested. Examination showed that all sensation was suspended, no pulsation of the heart could be felt, and there was only an occasional gasping respiration. Practically, the animal was dead, and would have remained so but for

* This operation is far preferable to the one recommended by Dalton, (Dalton's Treatise on Human Physiology, p. 120), in which the stomach is freely incised and secured to the canula by ligatures, from the fact that in Dalton's operation there is necessarily sloughing of the stomach tissues included in the ligatures, and subsequent exhausting leakage from the stomach, while in this operation there is no sloughing at all nor any considerable leakage. The animal exhibited, did not lose a day's rations of meat, nor a pound of flesh, as the result of the operation.

TRANSFUSION, which technically consists in passing the blood of one animal into the veins of another—or, as in this case, the returning of blood to the veins of an animal from which it had been drawn.

This is most readily accomplished by means of a syringe, having a closely-fitting plain joint between body and nozzle, rendering detachment easy—the nozzle to be supplied with a stop-cock to prevent the entrance of air into the veins while the syringe is being filled.

The nozzle having been adjusted in the jugular vein, as before described, the transfusion was commenced; about one ounce per minute being the rate of injection. At about the fifth ounce injected, the animal commenced to wink and move his eyes, as though observing those around him; then, as the operation proceeded, to move his limbs, wag his tail, and give attention when spoken to. Sixteen ounces of blood were returned to the animal in this manner; immediately after which he raised his head voluntarily and looked around, and, with very little assistance, supported himself upon his haunches.

Subsequently to this, air was injected into the animal's veins, for the purpose of demonstrating its results. The first and second syringefull (half-ounce syringe used) produced no perceptible effect. The third and fourth produced irregular action of heart, disturbance in respiration, general convulsive movements of body, which, however, subsided in a few minutes, and the vital functions seemed to proceed regularly, although the animal was exhausted by the experiment. Some five or six syringefulls were then injected rapidly, when the heart ceased to act, a few convulsive efforts to breathe were made, and death ensued.

REMARKS CONCERNING TRANSFUSION.

While the animal was being prepared for transfusion, as heretofore described, Prof. Freer referred briefly to the history of the operation about to be performed. He said: The first successful experiments in transfusion were performed by Dr. Lower, of Oxford University, England, about the middle of the seventeenth century. It consisted of connecting the carotid artery of one animal to the jugular vein of another by means of a tube; and whilst blood was thus transfused, he also permitted it to flow from time to time from a tube inserted into the upper portion of the jugular vein of the animal upon which transfusion was practiced, that as plethora ensued it might be reduced. Dr. Lower proposed to experiment to ascertain the effect of changing the blood of the old and young, the sick and healthy, and of the cold and warm blooded animals, expecting that this exchange would alter the nature of the animals experimented upon.

Transfusion was first performed upon the human subject in 1667, by two Frenchmen, Dennis and Emeretz, who made use of the blood of a sheep. The basilic vein of the young man was connected with the carotid artery of the sheep, and the blood thus transfused. The man survived the operation. The next experiment of these gentlemen was upon an insane man, calf's blood being used. The experiment continued two days, and over a pound of blood was transfused. At first the man complained of suffocation, and the perspiration started from him freely. At the end of the experiment he became sane and was restored to his family. Another experiment upon a man of sound mind was also tried with sheep's blood, and succeeded admirably. The result was hailed with great enthusiasm. It was believed that these experiments had discovered, so far as health and youth were concerned, the "Philosopher's Stone," so much sought after; that an old man could be rendered young, and a sick man well, by transfusion. But unluckily the insane man returned to his old mental condition, and died during a second operation. Parliament then, by special act, put an end to further experiments of the kind. After an interval of a century, the study of transfusion was resumed by Harwood, of Cambridge, England, who insisted upon the fact that in transfusion of blood among animals of different species they generally died some days after the operation.

With Blundell, Prevost, and Dumas, the subject of transfusion entered upon a new phase. It is above all due to the works of these laborers and those who followed, such as Dieffenbach, Thos. Bischoff, Brown Sequard, and others, that this subject has attained such interest, in a double point of view, to therapeutics and physiology.

Prof. Freer urged the necessity of defibrinating the blood; 1st, as a precaution against accident, and 2d, because by so doing the operation can be proceeded with without haste, and is, indeed, perfectly at the command of a skillful manipulator.

Prof. Freer then stated that transfusion had been performed twice on the human subject, once in this city, and once in Atlantic, Iowa, and requested Dr. W. C. Hunt to read the notes of the two cases, which were as follows, viz:

CASE I. Feb. 7, 1870. Drs. Hunt and Wadsworth, in compliance with a request by telegraph from Mr. G. P. S., of Atlantic, Iowa, went to that place to perform "transfusion" upon his daughter, 18 years of age, who was supposed to be rapidly sinking from a lack of nutrition, occasioned by the effects of a drachm of aqua ammonia, administered by mistake. This was believed by the parents to have produced so severe an inflammation of the stomach as to have "destroyed the mucous membrane."

Her previous history was as follows: Slight hereditary taint of scrofula on paternal side. Fifteen months prior to this date, she became somewhat anæmic; the menstrual flow was suspended, and she had gradually declined in health from that time. About two months since (in Dec., 1869) the ammonia was taken. This hastened her decline, and on or about the 25th of Jan., 1870, she contracted a severe cold, which compelled her to take to her bed.

At the time Drs. H. and W. saw her, her condition was excessively anæmic, with the muscular and adipose tissues much wasted.

Symptoms. Pulse very irregular, varying from 112 to 150 per minute, small, thready, and at times almost imperceptible at the wrist.

Respiration 22 per minute, imperfect and labored.

Severe lancinating pains, principally in the right lumbar and hypochondriac regions; countenance expressive of great suffering and anxiety.

There were some evidences of tubercle in the lungs, but the severe cold, under which she was laboring, masked them somewhat.

The patient seemed in such imminent danger of death that they hesitated to transfuse, fearing she would die under the operation, but, at the urgent solicitation of her parents, finally consented. Preparations were accordingly made, and the operation performed, as follows:

At 11 A. M., Feb. 8, anæsthetized patient and inserted a canula in the cephalic vein of left arm. At 11.45, drew from the arm of a brother of patient, vinous blood, which, when defibrinated, amounted to eleven ounces.

This was kept at a temperature of 100 deg. F., by means of a water bath.

At 12.45 P. M., patient having fully recovered from the anæsthesia—pulse 112 and irregular, respiration as before stated—commenced injecting blood. This was done very slowly. At 1.15 P. M. the eleven ounces had been thrown in. No unusual sensations nor pain were experienced by the patient until the 10th syringefull had been injected, when she voluntarily remarked that she "*felt better.*" From this time to the close of the operation she several times and in different ways expressed her great sense of relief from suffering and anxiety.

Pulse, at 1.15 P. M., 80 per minute, volume much increased and steadier. Respiration greatly improved, and she was much more quiet. The pain, before noted, continued, and the apparent improvement was not of long duration.

2.30 P. M. Pulse 100. The pain and restlessness continuing, at this time a hypodermic injection of morphia 1-4 gr., atropia 1-60 gr., was made.

In a few minutes, the pain being relieved, she sank into a profound sleep, which at 4.30 P. M. became comatose, and we became fearful that she would never be aroused. The lungs were filling, and the efforts of coughing were feeble and inefficient.

During the next hour, there being no change for the worse, we decided to inject more blood.

This time a sister of the patient furnished the blood—fourteen ounces (defibrinated.)

6.50 P. M. Pulse 132, irregular, feeble; respiration slow, imperfect; patient comatose. Commenced injecting. In 36 minutes had finished injecting the fourteen ounces.

7.26 P. M. Pulse 120. As much improved in every respect as in the previous operation. Respiration also better in every way, and patient apparently *sleeping quietly.*

At about 10 P. M., a recurrence of the sinking, such as she experienced a few hours before, occurred. Stimulants and nourishment were administered as before, and she gradually revived and regained her consciousness, but suffered from a return of the pain before described. This lasted during the whole of the night, as local applications only were made to relieve them.

Feb. 9, 7 A. M. Patient sinking; expresses a desire to have the operation again performed.

Dr. Wadsworth having returned home, Dr. Hunt, with the assistance of the attending physician, Dr. ——, consented to do it. Another brother of the patient furnished the blood (thirteen ounces, defibrinated.)

8.14 A. M. Pulse 130; other conditions as before. Respiration also as before. Patient rapidly sinking. Commenced injecting. In 21 minutes had finished (13 oz.) Pulse had fallen to 120, and together with the respiration were improved as markedly as in the two operations. The pain in the side continued as before.

Shortly after this time Dr. H. returned home, and in a couple of days learned that the patient gradually sank, and died on the afternoon of the same day.

The second case was a patient of Dr. P. Matthei, of Chicago, Mrs. S., aged 30 years. In the spring of 1870, she miscarried at the seventh month. Profuse hemorrhage accompanied and followed this at frequent irregular intervals, until the anæmia became alarming. Symptoms of septicaunia supervened, and, as a last hope, "transfusion" was suggested, and on the 21st of May—nearly three months after the occurrence of the abortion—it was decided to "*transfuse.*" Accordingly, with the following conditions and results, Drs. Hunt and Matthei performed the operation.

General condition of patient as above stated. Special condition: Pulse 132 per minute, small, thready, and at times imperceptible at the wrist. Respiration 58 per minute, spasmodic, gasping, sighing. Temperature in the axilla and mouth 97 deg. F. No delirium.

Preparations being made as in case 1, at 4 P. M. commenced injecting. In half-an-hour had injected nine ounces, pulse had fallen to 123, and, together with respiration, was improved in every respect. Temperature 98.

Patient expressed and otherwise manifested a great sense of relief from the exhaustion and anxiety from which she had so long suffered.

The change was so marked that the attendants observed and commented upon it.

Still, death was yet apparently so imminent, that the result of a few hours was waited for, with the intention of repeating the operation, but during the following morning she sank, and died ten hours after the operation.

Almost beyond question her life was prolonged for nearly this space of time by the "*transfusion.*"

